# Supplementary material for: Transmembrane channel-like 4 and 5 proteins at microvillar tips are potential ion channels and lipid scramblases
Source: bioRxiv. 2024 Aug 23:2024.08.22.609173. Preprint. [Version 1] doi: 10.1101/2024.08.22.609173 (PMC11370596; doi:10.1101/2024.08.22.609173)
Supplement: 1 [file NIHPP2024.08.22.609173v1-supplement-1.pdf]

bioRxiv preprint doi: https://doi.org/10.1101/2024.08.22.609173; this version posted August 23, 2024. The copyright holder for this preprint (which was not certified by peer review) is the author/funder. This article is a US Government work. It is not subject to copyright under 17 USC 105 and is also made available for use under a CC0 license.

Supplementary Materials

Transmembrane channel-like 4 and 5 proteins at microvillar tips are potential ion channels and lipid scramblases

Seham Ebrahim[#1], Angela Ballesteros[2,3], W. Sharon Zheng[1], Shounak Mukherjee[4], Gaizun Hu[1], Wei-Hsiang Weng[4,5,6], Jonathan S. Montgomery[4,7], Yaw Agyemang[4], Runjia Cui[2], Willy Sun[2], Evan Krystofiak[2], Mark P. Foster[4,5,7], Marcos Sotomayor[4,5,6,7], Bechara Kachar[2]

1) Center for Membrane and Cell Physiology, Department of Molecular Physiology and Biological Physics, University of Virginia, Charlottesville, VA 22903, USA.
2) Laboratory of Cell Structure and Dynamics, National Institute on Deafness and Other Communication Disorders, National Institutes of Health, Bethesda, MD 20892, USA.
3) Section on Sensory Physiology and Biophysics, National Institute on Deafness and Other Communication Disorders, National Institutes of Health, Bethesda, MD 20892, USA.
4) Department of Chemistry and Biochemistry, The Ohio State University, Columbus, OH 43210, USA.
5) Biophysics Program, The Ohio State University, Columbus, OH 43210, USA.
6) Department of Biochemistry and Molecular Biology, University of Chicago, Chicago, IL 60637, USA.
7) Ohio State Biochemistry Program, The Ohio State University, Columbus, OH 43210, USA.

Correspondence:
Seham Ebrahim, Ph.D.
Center for Membrane and Cell Physiology
Department of Molecular Physiology and Biological Physics
University of Virginia School of Medicine
Charottesville, VA 22903, USA.
e-mail : seham.ebrahim@virginia.edu

Bechara Kachar, M.D.
Laboratory of Cell Structure and Dynamics,
National Institute on Deafness and Other Communication Disorders, National Institutes of Health, Bethesda, MD 20892, USA.
e-mail : kacharb@nidcd.nih.gov

Running Title: TMC4/5 at microvillar tips

bioRxiv preprint doi: https://doi.org/10.1101/2024.08.22.609173; this version posted August 23, 2024. The copyright holder for this preprint (which was not certified by peer review) is the author/funder. This article is a US Government work. It is not subject to copyright under 17 USC 105 and is also made available for use under a CC0 license.

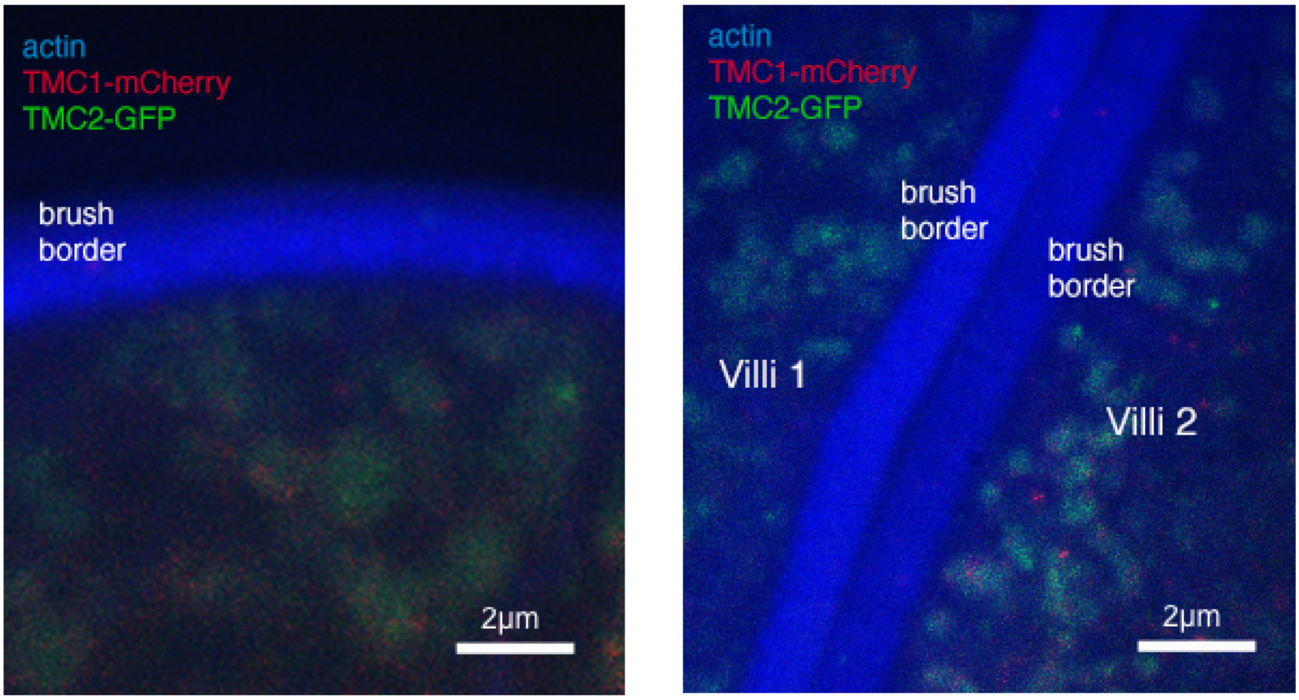

Figure S1: TMC1 and TMC2 are not expressed in IEC microvilli. Brush border microvilli stained with phalloidin (blue) in knockin mice expressing endogenous TMC2 tagged with GFP (green) and TMC1 tagged with mCherry (red)[1].

bioRxiv preprint doi: https://doi.org/10.1101/2024.08.22.609173; this version posted August 23, 2024. The copyright holder for this preprint (which was not certified by peer review) is the author/funder. This article is a US Government work. It is not subject to copyright under 17 USC 105 and is also made available for use under a CC0 license.

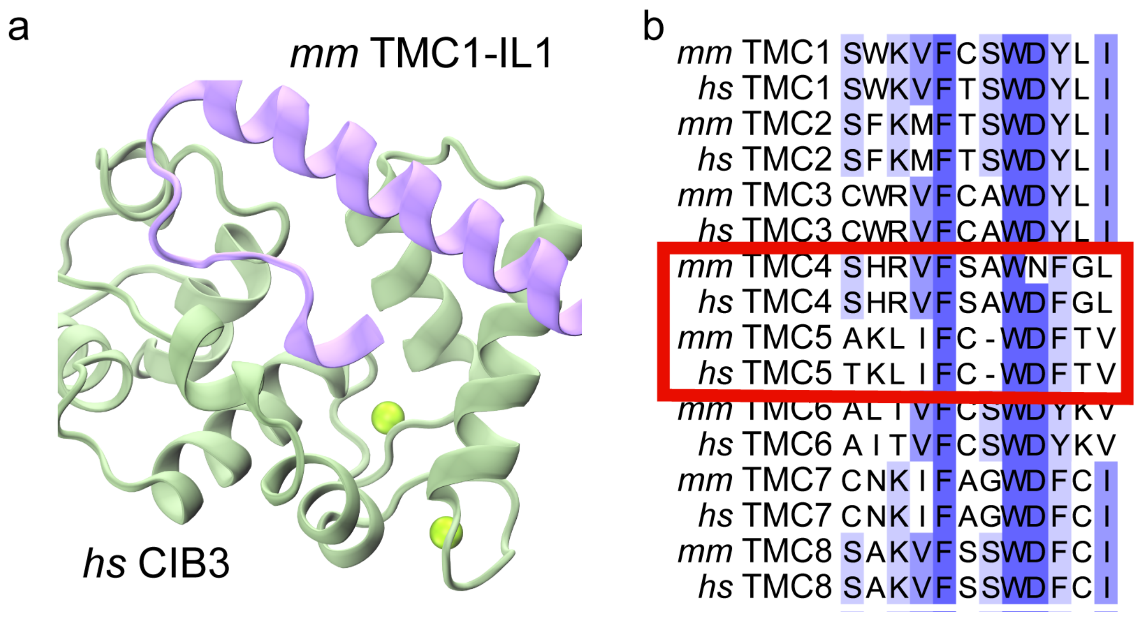

Figure S2: Conservation of TMC-IL1 and CIB interface. (a) Crystal structure of mouse (*Mus musculus*, *mm*) TMC1-IL1 and human (*Homo sapiens*, *hs*) CIB3 [PDB 6WUD][2]. (b) Multiple sequence alignment comparing sequences of the IL1 region of mouse and human TMC1 through TMC8 (NP_083229.1, NP_619636.2, NP_619596.1, NP_542789.2, NP_808363.3, NP_001074001.1, NP_861541.2, NP_001138775.2, NP_001098722.1, NP_001098718.1, NP_663414.3, NP_001120670.1, NP_766064.2, NP_001287661.1, NP_001182017.1, NP_689681.2). The sequence motif FXXWDF/Y is conserved. Alignment is colored by sequence similarity with white being the lowest similarity and blue being the highest. Some columns might not be colored if deletions or changes to residue type are present (e.g., polar amino acid to hydrophobic).

bioRxiv preprint doi: https://doi.org/10.1101/2024.08.22.609173; this version posted August 23, 2024. The copyright holder for this preprint (which was not certified by peer review) is the author/funder. This article is a US Government work. It is not subject to copyright under 17 USC 105 and is also made available for use under a CC0 license.

Supplementary Fig. 3

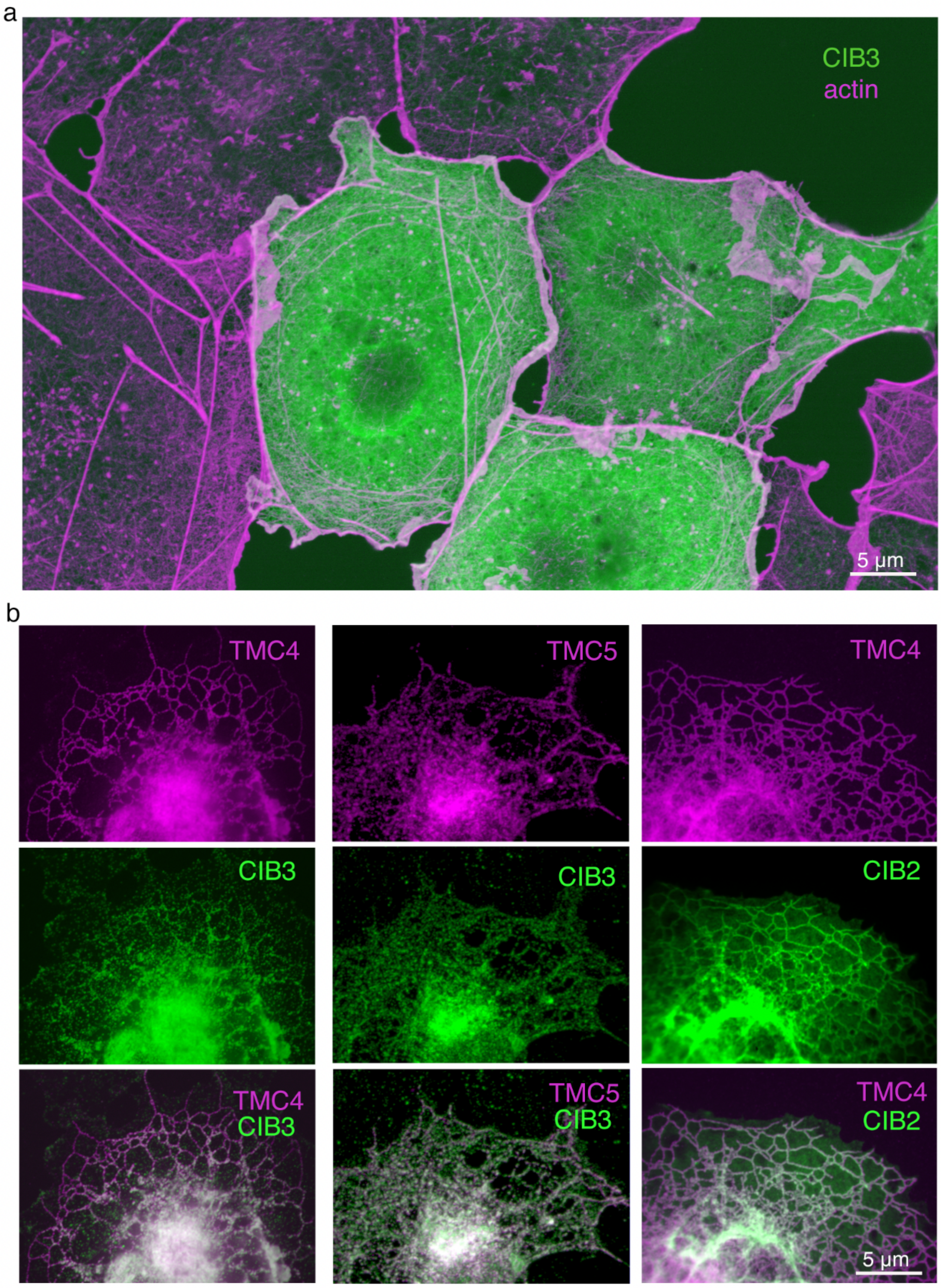

Figure S3. CIB family proteins colocalize with TMC4 and 5 in COS7 cells. (a) COS7 cells transfected with CIB3-DDK, labeled with anti-CIB3 antibody (green) and phalloidin to mark actin (magenta). (b) COS7 cells co-transfected with TMC4- or TMC5-mCherry (magenta) and CIB2- or CIB3-DDK, and immuno-labeled with anti-CIB3 antibody (green), or anti-DDK antibody for CIB2 (green).

bioRxiv preprint doi: https://doi.org/10.1101/2024.08.22.609173; this version posted August 23, 2024. The copyright holder for this preprint (which was not certified by peer review) is the author/funder. This article is a US Government work. It is not subject to copyright under 17 USC 105 and is also made available for use under a CC0 license.

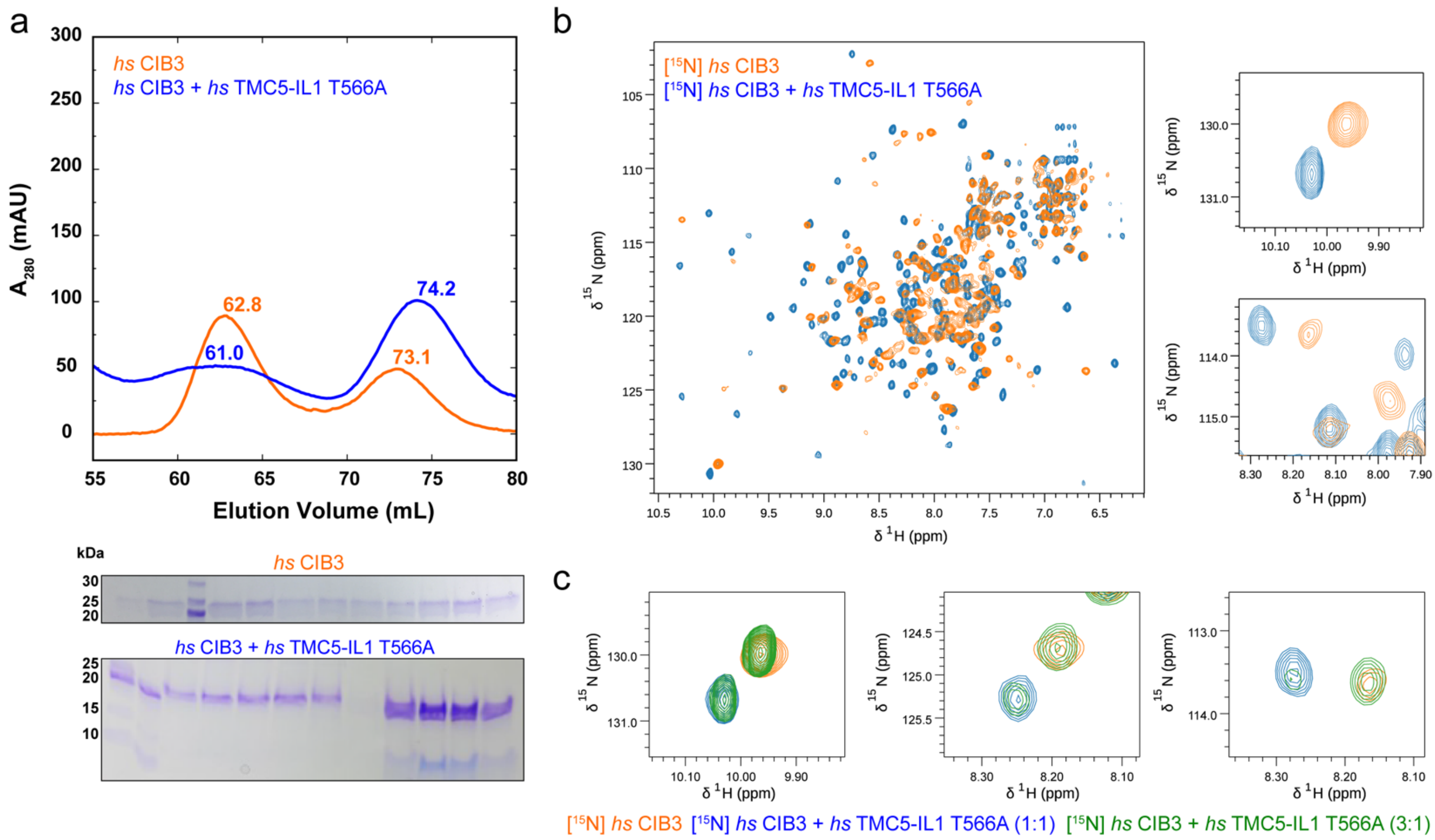

Figure S4. NMR data supports CIB3 and TMC5-IL1 complex formation. (a) Size exclusion chromatorgraphy (SEC) of CIB3 either refolded alone or co-refolded with *hs* TMC5-IL1 T566A (equivalent to *mm* TMC5-IL1). SEC curve and SDS-PAGE gel for CIB3 alone were adapted from Giese et al. (2024)[3]. (b) Overlay of ^{1}H-^{15}N TROSY-HSQC spectra of [U-^{15}N]-CIB3 alone (orange) and bound to TMC5-IL1 (blue). (c) Detail of overlay of ^{1}H-^{15}N TROSY-HSQC spectra of [U-^{15}N]-CIB3 alone (orange) and bound to TMC5-IL1 at 1:1 (blue) and 3:1 (green) CIB3:TMC5-IL1 ratios. Spectrum for [U-^{15}N]-CIB3 alone was adapted from Giese et al. (2024)[3].

bioRxiv preprint doi: https://doi.org/10.1101/2024.08.22.609173; this version posted August 23, 2024. The copyright holder for this preprint (which was not certified by peer review) is the author/funder. This article is a US Government work. It is not subject to copyright under 17 USC 105 and is also made available for use under a CC0 license.

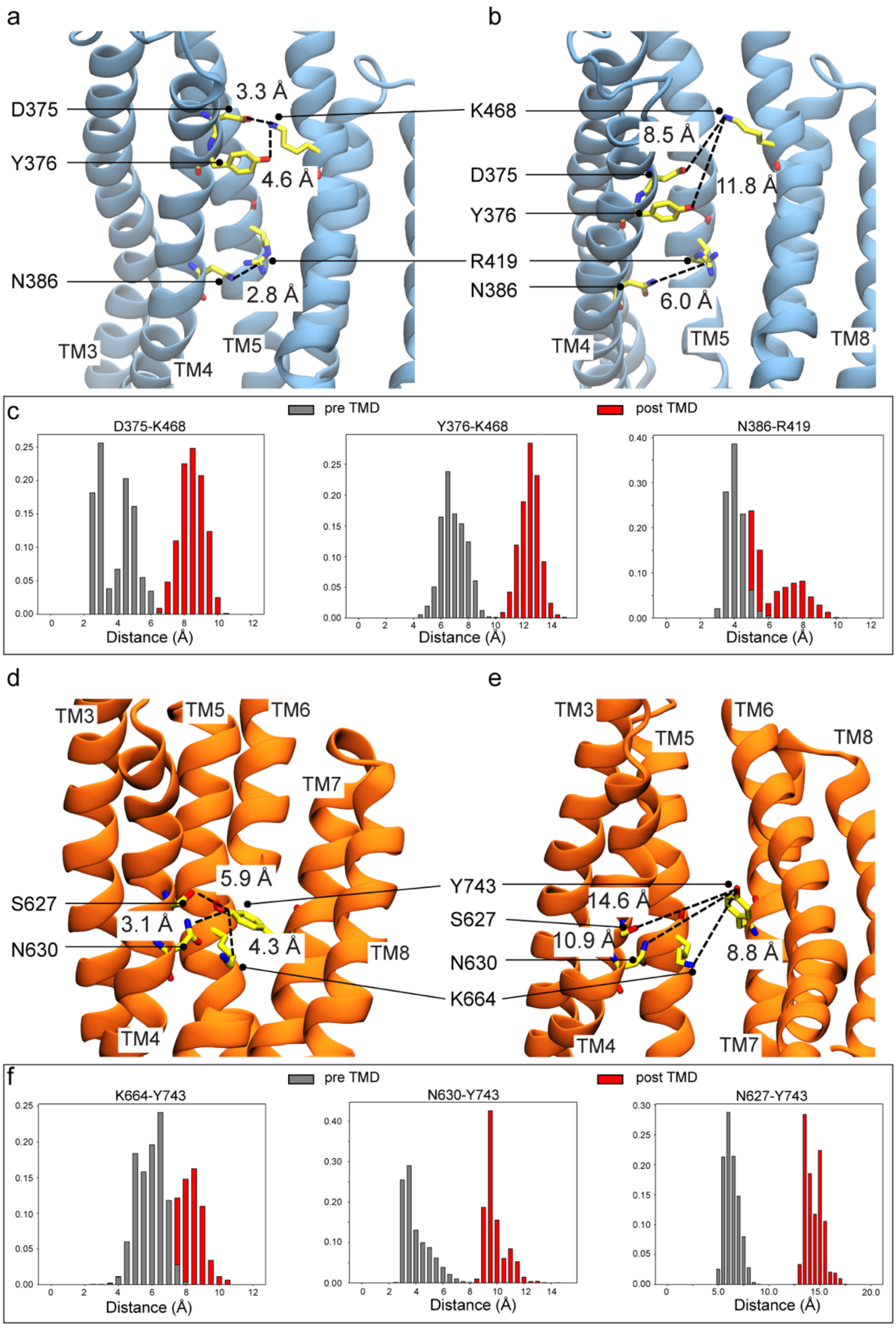

Figure S5. Closed and open pore models of TMC4 and 5. (a-b) AF2 and homology models representing closed and open pores for TMC4. Distances between D375 (TM4) – K468 (TM6), N386 (TM4) – R419 (TM5), and Y376 (TM4) – K468 (TM6) are shown to illustrate separation between TM4 and TM5/TM6 helices. (c) Distribution of distances between residues highlighted in (a) and (b) for the closed pore (pre TMD) and the open pore with constraints (post TMD). (d-e) AF2 and homology models representing closed and open pores for TMC5. Distances between S627 (TM4) – Y743 (TM7), N630 (TM4) – Y743 (TM7), and K664 (TM5) – Y743 (TM7) are shown to illustrate separation between TM7 and TM4/TM5 helices.

bioRxiv preprint doi: https://doi.org/10.1101/2024.08.22.609173; this version posted August 23, 2024. The copyright holder for this preprint (which was not certified by peer review) is the author/funder. This article is a US Government work. It is not subject to copyright under 17 USC 105 and is also made available for use under a CC0 license.

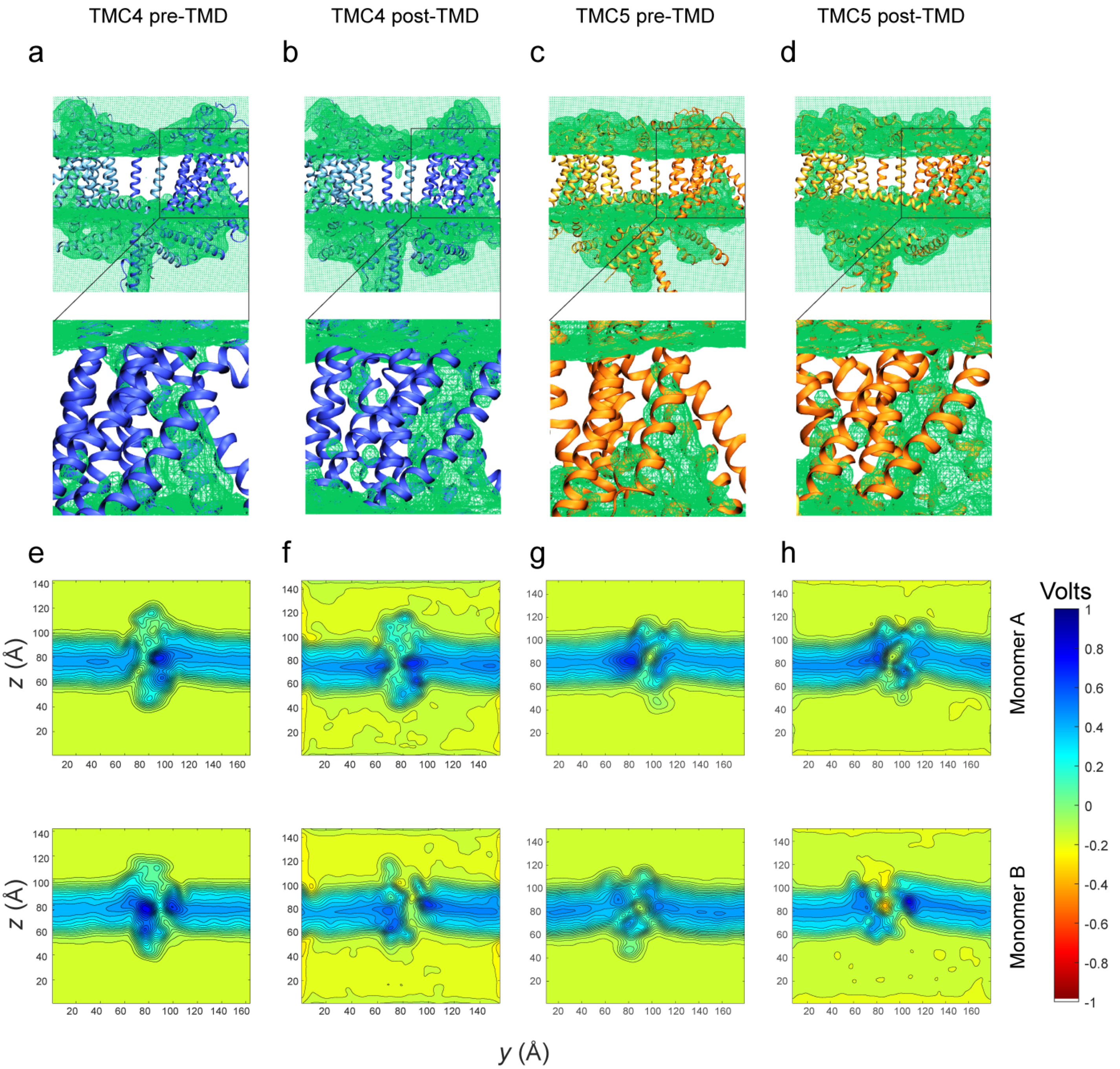

Figure S6. Water density and electrostatic maps for TMC4 and 5. (a-d) Water density maps for equilibration simulations of the TMC4 system prior (a) and post TMD (b), and of the TMC5 system prior (c) and post TMD (d). (e-f) Average electrostatic maps for TMC4 monomers A (top) and B (bottom), pre TMD (e) and post TMD (f) equilibrium simulations. (g-h) Average electrostatic maps for TMC5 monomers A (top) and B (bottom) pre (g) and post TMD (h). All post TMD simulations were performed with constraints on the backbone atoms of channel residues.

bioRxiv preprint doi: https://doi.org/10.1101/2024.08.22.609173; this version posted August 23, 2024. The copyright holder for this preprint (which was not certified by peer review) is the author/funder. This article is a US Government work. It is not subject to copyright under 17 USC 105 and is also made available for use under a CC0 license.

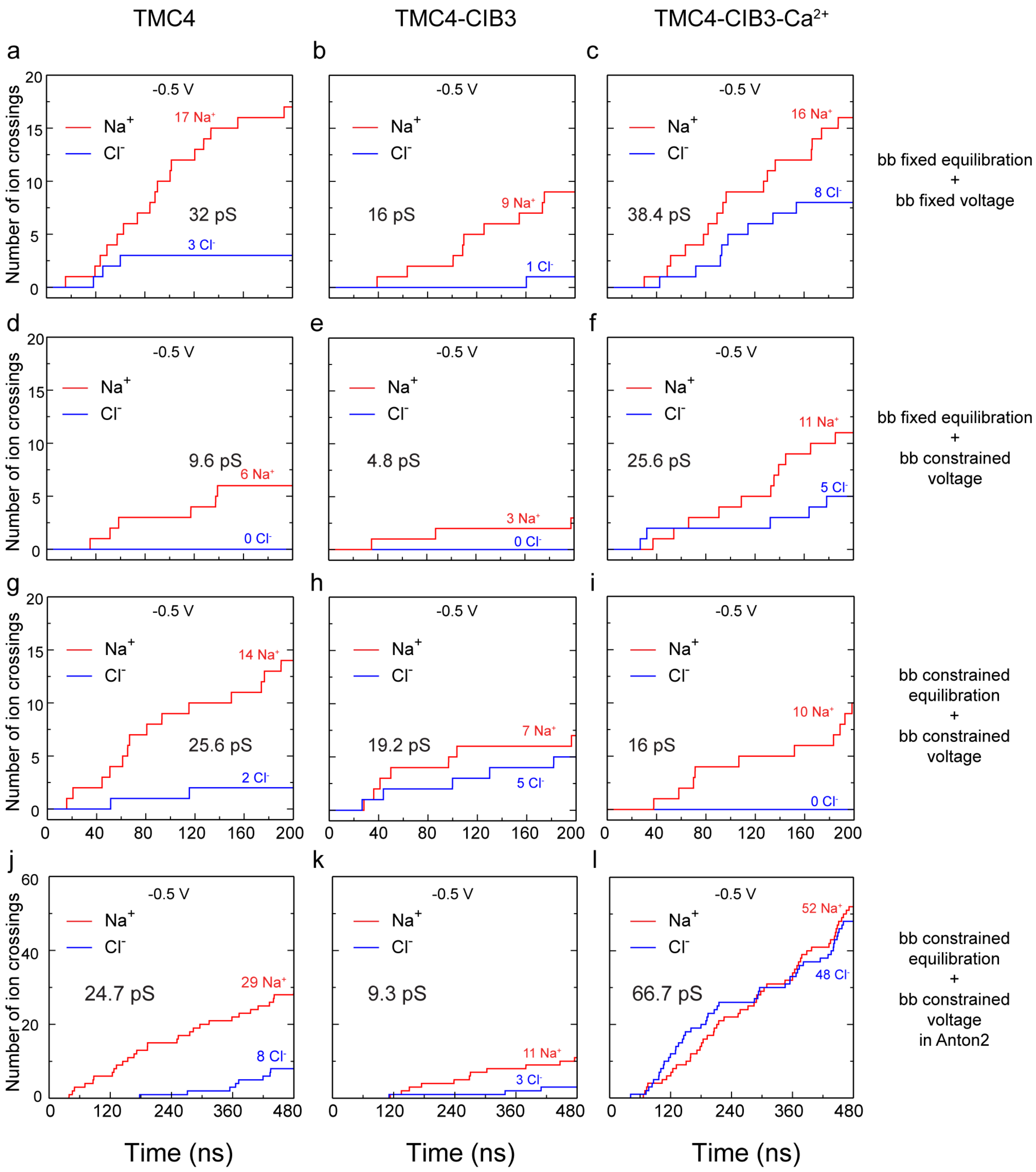

Figure S7. Number of ion crossings vs time for different simulations of TMC4, TMC4-CIB3, and TMC4-CIB3-Ca^{2+} systems. Number of ion crossings as a function of time for the conducting pore (chain B) of *mm* TMC4 after TMD was performed on chain B. Plots are shown for 200-ns long simulations at -0.5 V (a-i) and 480-ns long Anton2 simulations (j-l). Some panels are also in Fig. 9.

bioRxiv preprint doi: https://doi.org/10.1101/2024.08.22.609173; this version posted August 23, 2024. The copyright holder for this preprint (which was not certified by peer review) is the author/funder. This article is a US Government work. It is not subject to copyright under 17 USC 105 and is also made available for use under a CC0 license.

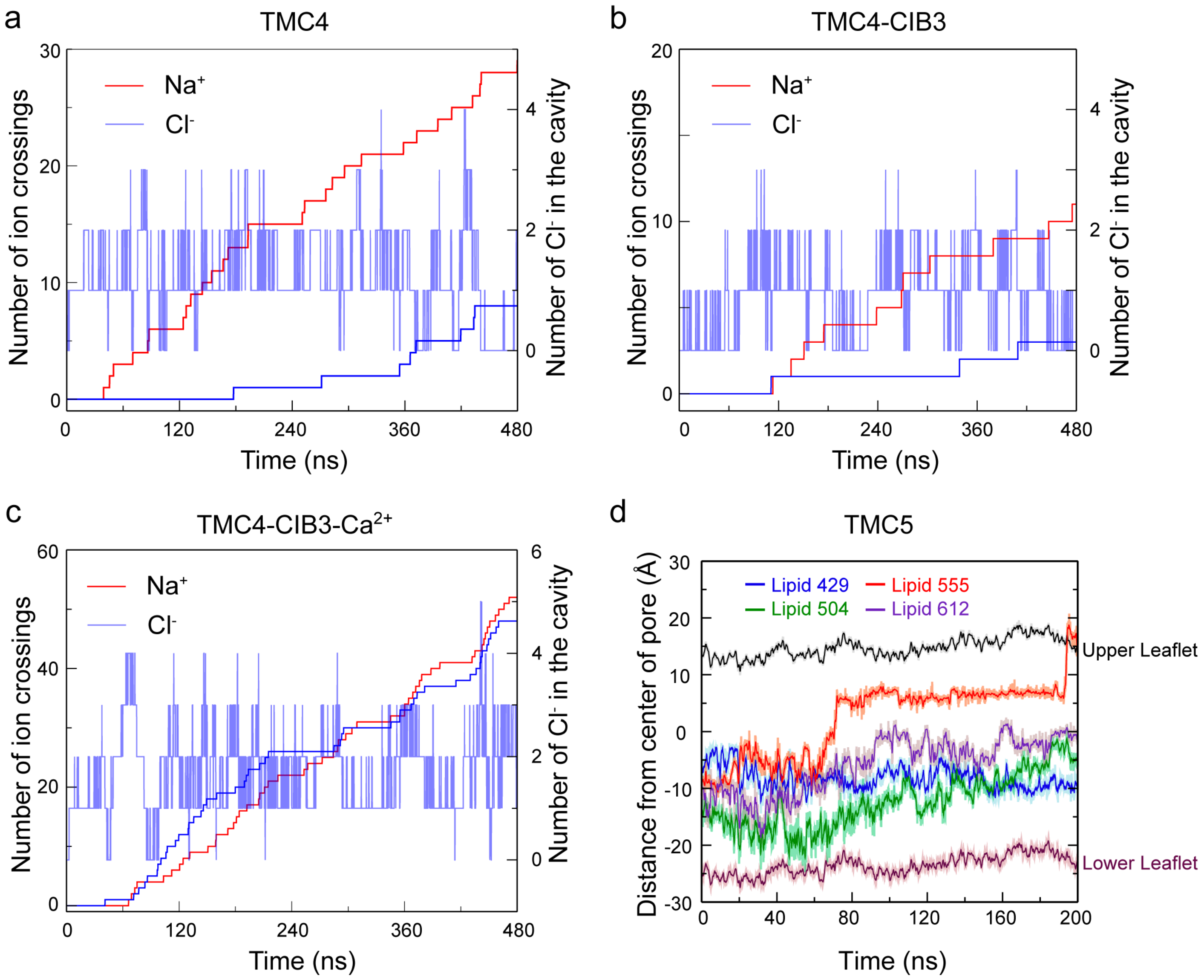

Figure S8. TMC4 and 5 pore properties. (a-c) Number of Na^+ and Cl^- ions crossing and Cl^- ions present inside the cavity at a certain point of time from simulations SA1m, SA2m, and SA3m (Table S2). An ion was considered to be inside the cavity if it had a *z*-coordinate in between (C_{cavity} +7 Å) and (C_{cavity} -13 Å) where C_{cavity} = *z*-coordinate of center of the pore defined by residues 320 to 560. (d) Plot showing the position of P atoms of lipid headgroups as a function of time with respect to the center of the channel in TMC5 at -0.5 V. Lipid 555 (red) is moving from the intracellular side towards the extracellular side. This trajectory is also shown in Fig. 9m.

bioRxiv preprint doi: https://doi.org/10.1101/2024.08.22.609173; this version posted August 23, 2024. The copyright holder for this preprint (which was not certified by peer review) is the author/funder. This article is a US Government work. It is not subject to copyright under 17 USC 105 and is also made available for use under a CC0 license.

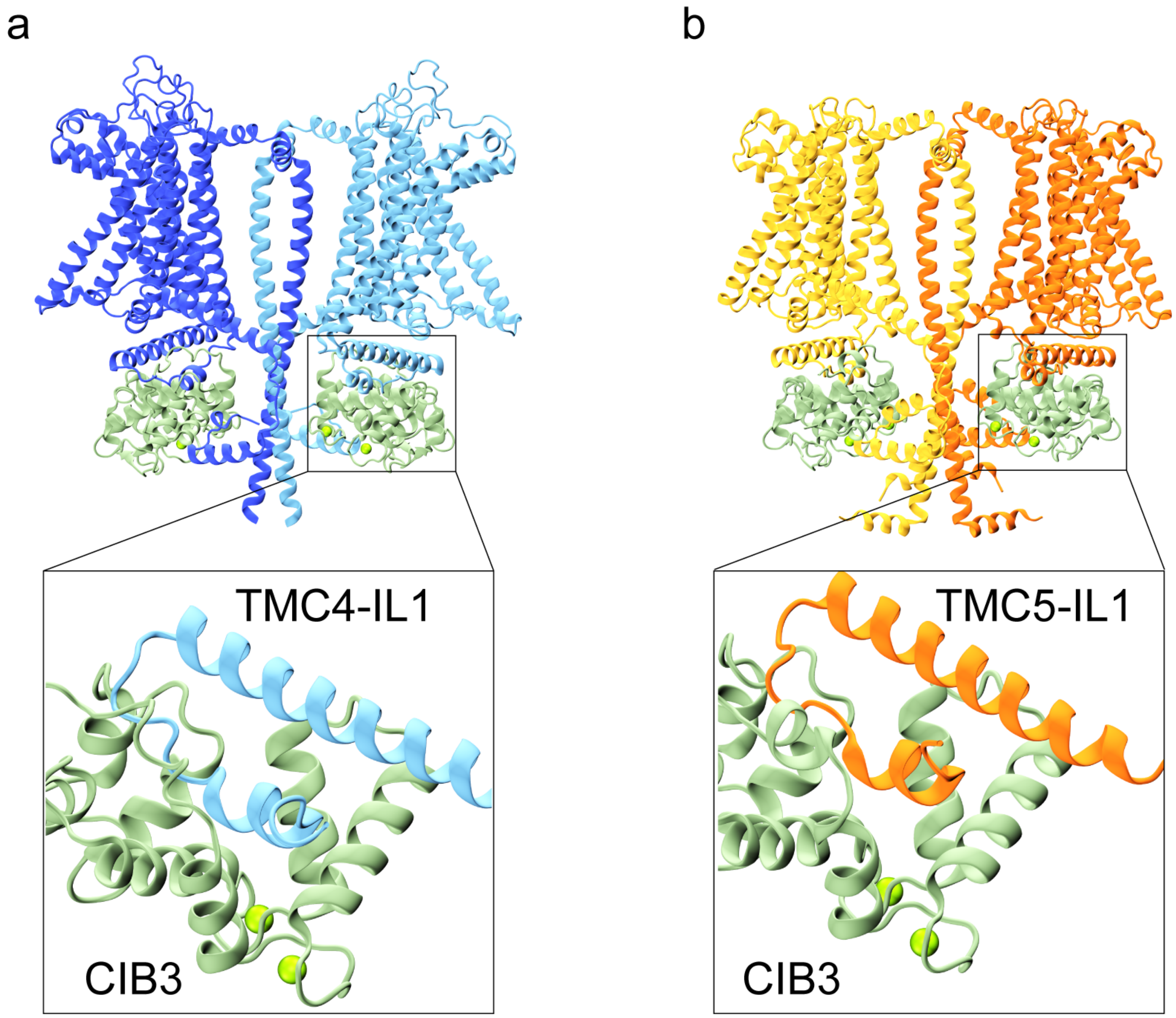

Figure S9. TMC and CIB interactions in AF2 models. (a-b) AF2 predicted models of CIB3 interacting with TMC4 (a) and 5 (b). Insets show details of the interaction with IL-1 for each protein. Ca^{2+} ions are shown as green spheres.

bioRxiv preprint doi: https://doi.org/10.1101/2024.08.22.609173; this version posted August 23, 2024. The copyright holder for this preprint (which was not certified by peer review) is the author/funder. This article is a US Government work. It is not subject to copyright under 17 USC 105 and is also made available for use under a CC0 license.

Table S1. Summary of Simulations

System	Label	Start	Voltage (V)	Length (ns)	Constrained region	Constraint type	# of atoms	Initial Size (nm^3)
mm TMC4	S1a	-	0	100	-	-	387,158	17.7 x 17.7 x 15.4
	S1b	S1a	-0.5	100	-	-		
	S1c	S1a	+0.5	100	-	-		
mm TMC4 (TMD)	S1d	S1a	0	10	-	-		
mm TMC4 (post TMD)	S1e	S1d	0	100	TM3-TM7	Fixed		
	S1f	S1e	-0.5	200	TM3-TM7	Fixed		
	S1g	S1e	+0.5	200	TM3-TM7	Fixed		
	S1h	S1e	-0.5	200	TM3-TM7	Harmonic		
	S1i	S1d	0	50	TM3-TM7	Harmonic		
	S1j	S1i	-0.5	200	TM3-TM7	Harmonic		
	S1k	S1i	+0.5	200	TM3-TM7	Harmonic		
	SA1l	S1d	0	120	TM3-TM7	Harmonic		
	SA1m	SA1l	-0.5	480	TM3-TM7	Harmonic		
mm TMC5	S2a	-	0	100	-	-	448,732	18.0 x 18.0 x 16.1
	S2b	S2a	-0.5	100	-	-		
	S2c	S2a	+0.5	100	-	-		
mm TMC5 (TMD)	S2d	S2a	0	10	-	-		
mm TMC5 (post TMD)	S2e	S2d	0	100	TM3-TM7	Fixed		
	S2f	S2e	-0.5	200	TM3-TM7	Fixed		
	S2g	S2e	+0.5	200	TM3-TM7	Fixed		
mm TMC5 (post TMD lipid deleted)	S2h†	S2f	0	10	TM3-TM7	Fixed	448,598	18.2 x 18.2 x 15.2
	S2i	S2h	-0.5	200	TM3-TM7	Fixed		
	S2j	S2h	-0.5	200	TM3-TM7	Harmonic		

bioRxiv preprint doi: https://doi.org/10.1101/2024.08.22.609173; this version posted August 23, 2024. The copyright holder for this preprint (which was not certified by peer review) is the author/funder. This article is a US Government work. It is not subject to copyright under 17 USC 105 and is also made available for use under a CC0 license.

mm TMC5 (post TMD)	SA2k	S2e	-0.5	480	TM3-TM7	Harmonic	448,732	16.9 x 17.3 x 15.1
mm TMC4-CIB3 w/o Ca2^{+}	S3a	-	0	100	-	-		
	S3b	S3a	-0.5	100	-	-		
	S3c	S3a	+0.5	100	-	-		
mm TMC4-CIB3 w/o Ca2^{+} (TMD)	S3d	S3a	0	10	-			
mm TMC4-CIB3 w/o Ca2^{+} (post TMD)	S3e	S3d	0	100	TM3-TM7	Fixed		
	S3f	S3e	-0.5	200	TM3-TM7	Fixed	404,672	16.5 x 16.5 x 15.8
	S3g	S3e	+0.5	200	TM3-TM7	Fixed		
	S3h	S3e	-0.5	200	TM3-TM7	Harmonic		
	S3i	S3d	0	50	TM3-TM7	Harmonic		
	S3j	S3i	-0.5	200	TM3-TM7	Harmonic		
	S3k	S3i	+0.5	200	TM3-TM7	Harmonic		
	SA3l	S3d	0	120	TM3-TM7	Harmonic		
	SA3m	SA3l	-0.5	480	TM3-TM7	Harmonic		
mm TMC4-CIB3-Ca2^{+}	S4a	-	0	100	-	-		
	S4b	S4a	-0.5	100	-	-		
	S4c	S4a	+0.5	100	-	-		
mm TMC4-CIB3-Ca2^{+} (TMD)	S4d	S4a	0	10	-	-		
mm TMC4-CIB3-Ca2^{+} (post TMD)	S4e	S4d	0	100	TM3-TM7	Fixed	404,660	16.5 x 16.5 x 15.8
	S4f	S4e	-0.5	200	TM3-TM7	Fixed		
	S4g	S4e	+0.5	200	TM3-TM7	Fixed		
	S4h	S4e	-0.5	200	TM3-TM7	Harmonic		
	S4i	S4d	0	50	TM3-TM7	Harmonic		
	S4j	S4i	-0.5	200	TM3-TM7	Harmonic		

bioRxiv preprint doi: https://doi.org/10.1101/2024.08.22.609173; this version posted August 23, 2024. The copyright holder for this preprint (which was not certified by peer review) is the author/funder. This article is a US Government work. It is not subject to copyright under 17 USC 105 and is also made available for use under a CC0 license.

	S4k	S4i	+0.5	200	TM3-TM7	Harmonic		
	SA4l	S4d	0	120	TM3-TM7	Harmonic		
	SA4m	SA4l	-0.5	480	TM3-TM7	Harmonic		
mm TMC5-CIB3 w/o $Ca2^+$	S5a	-	0	100	-	-	445,668	16.8 x 16.8 x 15.6
	S5b	S5a	-0.5	100	-	-		
	S5c	S5a	+0.5	100	-	-		
mm TMC5-CIB3 w/o $Ca2^+$ (TMD)	S5d	S5a	0	10	-	-		
mm TMC5-CIB3 w/o $Ca2^+$ (post TMD)	S5e	S5d	0	100	TM3-TM7	Fixed		
	S5f	S5e	-0.5	100	TM3-TM7	Fixed		
	S5g	S5e	+0.5	100	TM3-TM7	Fixed		
	S5h	S5d	0	100	TM3-TM7	Harmonic		
	S5i	S5h	-0.5	100	TM3-TM7	Harmonic		
	S5j	S5h	+0.5	100	TM3-TM7	Harmonic		
	S5Ak	S5h	-0.5	480	TM3-TM7	Harmonic		
mm TMC5-CIB3-$Ca2^+$	S6a	-	0	100	-	-	445,094	16.8 x 16.8 x 15.5
	S6b	S6a	-0.5	100	-	-		
	S6c	S6a	+0.5	100	-	-		
mm TMC5-CIB3-$Ca2^+$ (TMD)	S6d	S6a	0	10	-	-		
mm TMC5-CIB3-$Ca2^+$ (post TMD)	S6e	S6d	0	100	TM3-TM7	Fixed		
	S6f	S6e	-0.5	100	TM3-TM7	Fixed		
	S6g	S6e	+0.5	100	TM3-TM7	Fixed		
	S6h	S6d	0	100	TM3-TM7	Harmonic		
	S6i	S6h	-0.5	200	TM3-TM7	Harmonic		

bioRxiv preprint doi: https://doi.org/10.1101/2024.08.22.609173; this version posted August 23, 2024. The copyright holder for this preprint (which was not certified by peer review) is the author/funder. This article is a US Government work. It is not subject to copyright under 17 USC 105 and is also made available for use under a CC0 license.

S6j	S6h	+0.5	100	TM3-TM7	Harmonic
SA6k	S6h	-0.5	480	TM3-TM7	Harmonic

† = Lipid that was stuck in the channel was deleted and a short minimization and equilibration was performed.

SA = Simulations performed using Anton2.

bioRxiv preprint doi: https://doi.org/10.1101/2024.08.22.609173; this version posted August 23, 2024. The copyright holder for this preprint (which was not certified by peer review) is the author/funder. This article is a US Government work. It is not subject to copyright under 17 USC 105 and is also made available for use under a CC0 license.

Table S2. Summary of Ion Conduction Events

Label	Voltage (V)	Length (ns)	Constrained region	Constraint type	Na+ (A)	Na+ (B)	Cl- (A)	Cl- (B)	I (pA)	C (pS)
S1a	0	100	-	-	-	-	-	-	-	-
S1b	-0.5	100	-	-	0	0	0	0	0	0
S1c	+0.5	100	-	-	0	0	0	0	0	0
S1d	0	10	-	-	-	-	-	-	-	-
S1e	0	100	TM3-TM7	Fixed	-	-	-	-	-	-
S1f	-0.5	200	TM3-TM7	Fixed	0	17	0	3	16	32
S1g	+0.5	200	TM3-TM7	Fixed	0	2	0	3	4	8
S1h	-0.5	200	TM3-TM7	Harmonic	0	6	0	0	4.8	9.6
S1i	0	50	TM3-TM7	Harmonic	-	-	-	-	-	-
S1j	-0.5	200	TM3-TM7	Harmonic	0	14	0	2	12.8	25.6
S1k	+0.5	200	TM3-TM7	Harmonic	0	4	0	0	3.2	6.4
SA1l	0	120	TM3-TM7	Harmonic	-	-	-	-	-	-
SA1m	-0.5	480	TM3-TM7	Harmonic	0	29	0	8	12.3	24.7
S2a	0	100	-	-	-	-	-	-	-	-
S2b	-0.5	100	-	-	0	0	0	0	0	0
S2c	+0.5	100	-	-	0	0	0	0	0	0
S2d	0	10	-	-	-	-	-	-	-	-
S2e	0	100	TM3-TM7	Fixed	-	-	-	-	-	-
S2f	-0.5	200	TM3-TM7	Fixed	0	0	0	0	0	0
S2g	+0.5	200	TM3-TM7	Fixed	0	0	0	0	0	0
S2h[†]	0	10	TM3-TM7	Fixed	-	-	-	-	-	-
S2i	-0.5	200	TM3-TM7	Fixed	0	4	0	10	11.2	22.4
S2j	-0.5	200	TM3-TM7	Harmonic	0	7	0	12	15.2	30.4

bioRxiv preprint doi: https://doi.org/10.1101/2024.08.22.609173; this version posted August 23, 2024. The copyright holder for this preprint (which was not certified by peer review) is the author/funder. This article is a US Government work. It is not subject to copyright under 17 USC 105 and is also made available for use under a CC0 license.

SA2k	-0.5	480	TM3-TM7	Harmonic	0	0	0	0	0	0
S3a	0	100	-	-	-	-	-	-	-	-
S3b	-0.5	100	-	-	0	0	0	0	0	0
S3c	+0.5	100	-	-	0	0	0	0	0	0
S3d	0	10	-	-	-	-	-	-	-	-
S3e	0	100	TM3-TM7	Fixed	-	-	-	-	-	-
S3f	-0.5	200	TM3-TM7	Fixed	0	9	0	1	8	16
S3g	+0.5	200	TM3-TM7	Fixed	0	1	0	0	0.8	1.6
S3h	-0.5	200	TM3-TM7	Harmonic	0	3	0	0	2.4	4.8
S3i	0	50	TM3-TM7	Harmonic	-	-	-	-	-	-
S3j	-0.5	200	TM3-TM7	Harmonic	0	7	0	5	9.6	19.2
S3k	+0.5	200	TM3-TM7	Harmonic	0	2	0	3	4	8
SA3l	0	120	TM3-TM7	Harmonic	-	-	-	-	-	-
SA3m	-0.5	480	TM3-TM7	Harmonic	0	11	0	3	4.7	9.3
S4a	0	100	-	-	-	-	-	-	-	-
S4b	-0.5	100	-	-	0	0	0	0	0	0
S4c	+0.5	100	-	-	0	0	0	0	0	0
S4d	0	10	-	-	-	-	-	-	-	-
S4e	0	100	TM3-TM7	Fixed	-	-	-	-	-	-
S4f	-0.5	200	TM3-TM7	Fixed	0	16	0	8	19.2	38.4
S4g	+0.5	200	TM3-TM7	Fixed	0	0	0	0	0	0
S4h	-0.5	200	TM3-TM7	Harmonic	0	11	0	5	12.8	25.6
S4i	0	50	TM3-TM7	Harmonic	-	-	-	-	-	-
S4j	-0.5	200	TM3-TM7	Harmonic	0	10	0	0	8	16
S4k	+0.5	200	TM3-TM7	Harmonic	0	0	0	0	0	0

bioRxiv preprint doi: https://doi.org/10.1101/2024.08.22.609173; this version posted August 23, 2024. The copyright holder for this preprint (which was not certified by peer review) is the author/funder. This article is a US Government work. It is not subject to copyright under 17 USC 105 and is also made available for use under a CC0 license.

SA4l	0	120	TM3-TM7	Harmonic	-	-	-	-	-	-
SA4m	-0.5	480	TM3-TM7	Harmonic	0	52	0	48	33.3	66.7
S5a	0	100	-	-	-	-	-	-	-	-
S5b	-0.5	100	-	-	0	0	0	0	0	0
S5c	+0.5	100	-	-	0	0	0	0	0	0
S5d	0	10	-	-	-	-	-	-	-	-
S5e	0	100	TM3-TM7	Fixed	-	-	-	-	-	-
S5f	-0.5	100	TM3-TM7	Fixed	0	0	0	0	0	0
S5g	+0.5	100	TM3-TM7	Fixed	0	0	0	0	0	0
S5h	0	100	TM3-TM7	Harmonic	-	-	-	-	-	-
S5i	-0.5	200	TM3-TM7	Harmonic	0	0	0	0	0	0
S5j	+0.5	100	TM3-TM7	Harmonic	0	1	0	0	1.6	3.2
SA5k	-0.5	480	TM3-TM7	Harmonic	0	9	0	8	5.67	11.34
S6a	0	100	-	-	-	-	-	-	-	-
S6b	-0.5	100	-	-	0	0	0	0	0	0
S6c	+0.5	100	-	-	0	0	0	0	0	0
S6d	0	10	-	-	-	-	-	-	-	-
S6e	0	100	TM3-TM7	Fixed	-	-	-	-	-	-
S6f	-0.5	100	TM3-TM7	Fixed	0	0	0	0	0	0
S6g	+0.5	100	TM3-TM7	Fixed	0	0	0	0	0	0
S6h	0	100	TM3-TM7	Harmonic	-	-	-	-	-	-
S6i	-0.5	200	TM3-TM7	Harmonic	0	1	0	1	1.6	3.2
S6j	+0.5	100	TM3-TM7	Harmonic	0	0	0	0	0	0
SA6k	-0.5	480	TM3-TM7	Harmonic	0	0	0	0	0	0

bioRxiv preprint doi: https://doi.org/10.1101/2024.08.22.609173; this version posted August 23, 2024. The copyright holder for this preprint (which was not certified by peer review) is the author/funder. This article is a US Government work. It is not subject to copyright under 17 USC 105 and is also made available for use under a CC0 license.

Movie Captions

Movie 1. mmTMC4-EQ.mp4. Chloride ions (green spheres) entering the TMC4 channel from the intracellular side during 100 ns equilibration simulation (Simulation S1a in Tables S1 and S2). Chain B of the protein is shown in light blue new cartoon representation in foreground while chain A has been shown in dark blue quicksurf representation. Chloride ions entering the channel oscillate among three basic residues R419 (TM5), R488 (TM6) and K549 (TM8) represented by yellow licorice with blue terminals (Nitrogen atoms). Water and lipid molecules have been omitted for visualization purposes.

Movie 2. mmTMC4-TMD.mp4. Targeted MD simulation showing the opening of chain B (light blue new cartoon) in TMC4 (Simulation S1d in Tables S1 and S2). TM4 slowly moves away from TM6, increasing the pore size. Polar residues are represented by yellow licorice. Blue quicksurf represents chain A. Water and lipid molecules have been omitted for visualization purposes.

Movie 3. mmTMC4-postTMD-conduction.mp4. Post-TMD ion crossings through chain B of TMC4, in the presence of -0.5 V (Simulation S1f in Tables S1 and S2). Chain B of TMC4 is shown using light blue new cartoon representation and chain A using blue quicksurf. Phosphorous atoms of lipid headgroups are shown using white spheres. Chloride ions (green spheres) cross from the intracellular to the extracellular side whereas sodium ions (yellow spheres) cross from the extracellular to the intracellular side. Lipid headgroups line up the periphery of the channel from both sides facilitating ion movement. Water molecules and lipid tails have been omitted for visualization purposes.

Movie 4. mmTMC5-lipid-flipping.mp4. Lipid flipping through chain B of TMC5 at -0.5 V (Simulations S2j in Tables S1 and S2). Chain B of TMC5 is shown using orange new cartoon representation in the foreground and chain A using dark yellow quicksurf representation. The phosphorous atoms of lipid headgroups are represented by white spheres. Lipid 555, represented by red spheres, undergoes flipping from intracellular to extracellular leaflet. Water, ions, and lipid tails have been omitted for visualization purposes.

Movie 5. mmTMC4-CIB3-Ca2+-conduction.mp4. Post-TMD ion crossings through chain B of the TMC4 in TMC4-CIB3-Ca2+ system at -0.5 V (Simulation S4f in Tables S1 and S2). Chain B of protein is shown using light blue new cartoon representation whereas chain A is shown using blue quicksurf. Phosphorous atoms of lipid headgroups are represented using white spheres. Chloride and sodium ions are represented using green and yellow spheres, respectively. We see more chloride ions crossing the channel in this system compared to only TMC4 (Movie 3). Water and lipid molecules have been omitted for visualization purposes.

bioRxiv preprint doi: https://doi.org/10.1101/2024.08.22.609173; this version posted August 23, 2024. The copyright holder for this preprint (which was not certified by peer review) is the author/funder. This article is a US Government work. It is not subject to copyright under 17 USC 105 and is also made available for use under a CC0 license.

References:

1. Kurima, K. *et al.* TMC1 and TMC2 Localize at the Site of Mechanotransduction in Mammalian Inner Ear Hair Cell Stereocilia. *Cell Rep.* **12**, 1606–1617 (2015).
2. Liang, X. *et al.* CIB2 and CIB3 are auxiliary subunits of the mechanotransduction channel of hair cells. *Neuron* **109**, 2131-2149.e15 (2021).
3. Giese, A. P. J. *et al.* Complexes of vertebrate TMC1/2 and CIB2/3 proteins form hair-cell mechanotransduction cation channels. *eLife* **12**, (2023).
